# Bilateral oculomotor nerve palsy secondary to bacterial meningitis

**DOI:** 10.1590/0037-8682-0564-2022

**Published:** 2023-03-06

**Authors:** Leonardo Kami, Bernardo Correa de Almeida Teixeira

**Affiliations:** 1 Universidade Federal do Paraná, Hospital de Clínicas, Departamento de Radiologia e Diagnóstico por Imagem, Curitiba, PR, Brasil.

**FIGURE 1:**
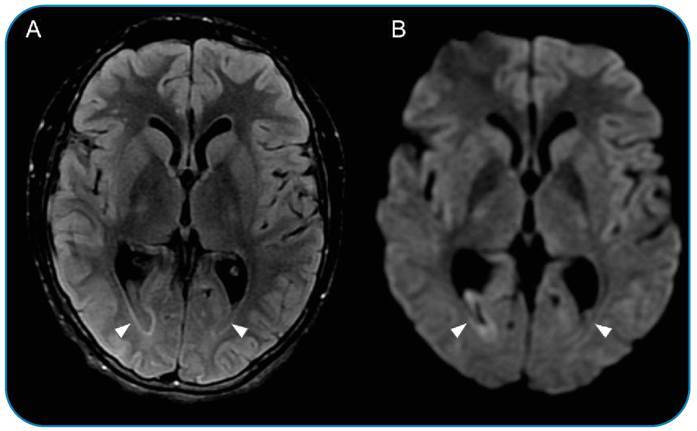
Magnetic resonance images of the brain show intermediate signal content in the lateral ventricles at FLAIR sequence **(A)** with restricted diffusion at DWI **(B)**.

**FIGURE 2:**
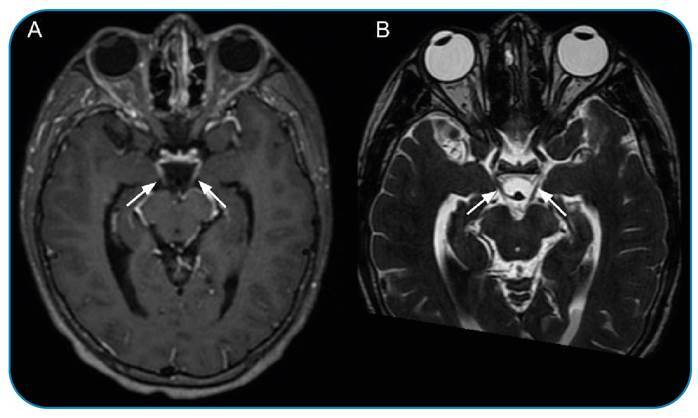
Magnetic resonance images of the brain show bilateral enhancement and thickening of the cisternal segments of the oculomotor nerves at T1-weighted contrast-enhanced **(A)** and high-resolution T2-weighted **(B)** sequences. Lateral deviation of the right eyeball can be observed.

A 17-year-old male was referred to a tertiary hospital with a 3-day history of headache, fever, neck stiffness, and progressive loss of consciousness. During hospitalization, he presented with bilateral mydriasis with fixed pupils, palpebral ptosis, and right horizontal gaze palsy. Cerebrospinal fluid analysis revealed xanthochromia, pleocytosis (280 WBC/microL), decreased glucose (<5.0 mg/dL), elevated protein (568.0 mg/dL), elevated lactate (16.1 mmoL/L), and latex agglutination positive for *S. pneumoniae.* Moreover, magnetic resonance imaging of the brain revealed intermediate signal content in the posterior segments of the lateral ventricles on FLAIR, with restricted diffusion on DWI, suggesting a dense/high protein content (Figure 1). Clinically, the most common presentation was purulent/inflammatory discharge or blood. However, in the T2* sequence, no low signals were observed, making suppurative inflammatory material secondary to ventriculitis highly likely. Leptomeningeal enhancement and thickening of the oculomotor nerves were also observed (Figure 2). After intensive care treatment with antibiotics and glucocorticoids, the patient was discharged with partial recovery from focal neurological deficits.

Cranial nerve palsies are a relatively uncommon complication of bacterial meningitis, occurring in approximately 4-11% of cases^1^, and are associated with a poor prognosis when presented during hospitalization^2^. The most common pathogen responsible is *S. pneumoniae* ^1^ ^,^ ^3^. Furthermore, the most affected cranial nerves are the oculomotor, abducens, facial, and trochlear nerves, and mainly the latter, because of sensitivity to high intracranial pressure. The pathophysiological explanation is nerve compression caused by pressure on the peripheral nerve, and perineuritis caused by meningeal inflammation.
